# Na_v _1.8-null mice show stimulus-dependent deficits in spinal neuronal activity

**DOI:** 10.1186/1744-8069-2-5

**Published:** 2006-02-14

**Authors:** Elizabeth A Matthews, John N Wood, Anthony H Dickenson

**Affiliations:** 1 Department of Pharmacology, University College London, Gower Street, London WC1E 6BT, UK; 2 Department of Biology, University College London, Gower Street, London WC1E 6BT, UK

## Abstract

**Background:**

The voltage gated sodium channel Na_v _1.8 has a highly restricted expression pattern to predominantly nociceptive peripheral sensory neurones. Behaviourally Na_v _1.8-null mice show an increased acute pain threshold to noxious mechanical pressure and also deficits in inflammatory and visceral, but not neuropathic pain. Here we have made in vivo electrophysiology recordings of dorsal horn neurones in intact anaesthetised Na_v _1.8-null mice, in response to a wide range of stimuli to further the understanding of the functional roles of Na_v _1.8 in pain transmission from the periphery to the spinal cord.

**Results:**

Na_v _1.8-null mice showed marked deficits in the coding by dorsal horn neurones to mechanical, but not thermal, -evoked responses over the non-noxious and noxious range compared to littermate controls. Additionally, responses evoked to other stimulus modalities were also significantly reduced in Na_v _1.8-null mice where the reduction observed to pinch > brush. The occurrence of ongoing spontaneous neuronal activity was significantly less in mice lacking Na_v _1.8 compared to control. No difference was observed between groups in the evoked activity to electrical activity of the peripheral receptive field.

**Conclusion:**

This study demonstrates that deletion of the sodium channel Na_v _1.8 results in stimulus-dependent deficits in the dorsal horn neuronal coding to mechanical, but not thermal stimuli applied to the neuronal peripheral receptive field. This implies that Na_v _1.8 is either responsible for, or associated with proteins involved in mechanosensation.

## Background

Voltage -gated sodium channels (VGSCs) are critical to action potential generation and neuronal excitability. Within the nervous system, at least nine subtypes, defined by their pore-forming α-subunit, each with individual functional and expression characteristics, are expressed [1]. Of these, Na_v _1.8 has a n expression pattern highly restricted to peripheral sensory neurones only, of which 85% or more respond to various painful or noxious stimuli [2,3]. Despite ongoing attempts to identify VGSC subtype-specific ligands, currently no agents exist that blocks the activity of Na_v _1.8 exclusively, thus its physiological role cannot be studied in vivo by pharmacological means. Generation of a Na_v _1.8 knockout mouse has provided an alternative solution to this problem and behaviourally these animals appear similar to littermate controls in terms of thresholds to non-noxious mechanical and noxious thermal stimulation. However, mice lacking Na_v _1.8 do show an increased acute pain threshold to noxious mechanical pressure and also deficits in inflammatory and visceral, but not neuropathic pain [4,5].

Behavioural assessment of the physiological function of channels or proteins implicated in pain pathways is extremely useful, but inherently does possess limitations. Nociceptive thresholds are commonly indicated by hindpaw withdrawal, thus preventing assessment of supra-threshold stimuli intensities. These latter responses represent the intensities of pain that are encountered in the clinic. The withdrawal response is further, not solely under the control of the sensory system. It is also dependent on the motor system and affected by motivation and sedation which may be altered in transgenic mice where the target gene is not exclusive to sensory pathways. Further, it has also been demonstrated that animal behavioural responses are influenced by many environmental factors [6,7]. For these reasons we have used in vivo electrophysiology recordings in intact anaesthetised Na_v _1.8-null mice, to record evoked activity of single dorsal horn neurones to a wide range of electrical and natural stimuli, which extends the limited neuronal characterisation of the phenotype from only electrical stimulation in vivo [4]. This technique allows characterisation of neuronal responses to a full range of modalities and intensities, including suprathreshold stimuli, focusing predominantly on the sensory pathways in a manner relatively independent of influencing environmental factors, to further to the understanding of the functional role of Na_v _1.8 in pain transmission.

## Results

Recordings were made from a total of 75 wide dynamic range (WDR) neurones in Na_v _1.8 -/- (*n *= 30) and littermate control Na_v _1.8 +/+ (*n *= 45) mice. The mean depths from the surface of the spinal cord of the neurones were 673 ± 17 μm and 561 ± 26 μm respectively, corresponding to the deep laminae. No difference was observed in the peripheral receptive field area of the spinal neurones in Na_v _1.8 +/+ (28.67 ± 2.57 % of total hindpaw area) and Na_v _1.8 -/- (24.65 ± 2.49 %) mice.

**Figure 1:**
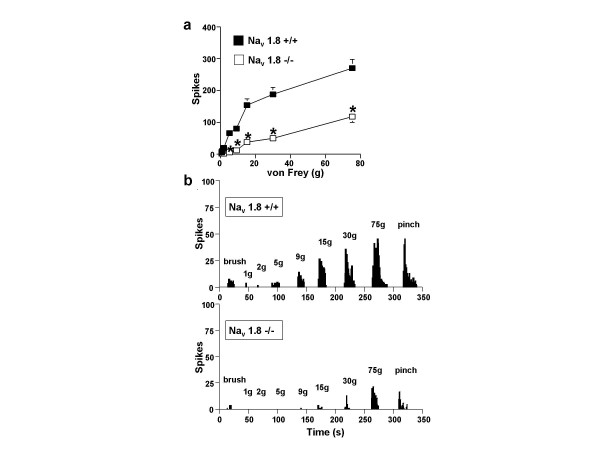
**Na_v _1.8-null mice display marked deficits in mechanically-evoked dorsal horn neuronal activity**. (a) Wide dynamic range neurones recorded from Na_v _1.8 -/- (n = 30) show significantly reduced activity to punctate mechanical von Frey stimuli compared to littermate control Na_v _1.8 +/+ (n = 45) mice. Data is expressed as means + or - sem. *p < 0.05. (b) Example traces of two single neurones, one recorded from a Na_v _+/+ mouse and one from a Na_v _-/- mouse. Stimuli were applied for 10 s onto the peripheral receptive field of the neurone located on the hindpaw. In both groups a graded response is displayed to increasing stimulus intensity (brush, von Frey 1 – 75 g and pinch), but this is markedly reduced in Na_v _1.8 -/- mice compared to Na_v _1.8 +/+ mice.

**Figure 2:**
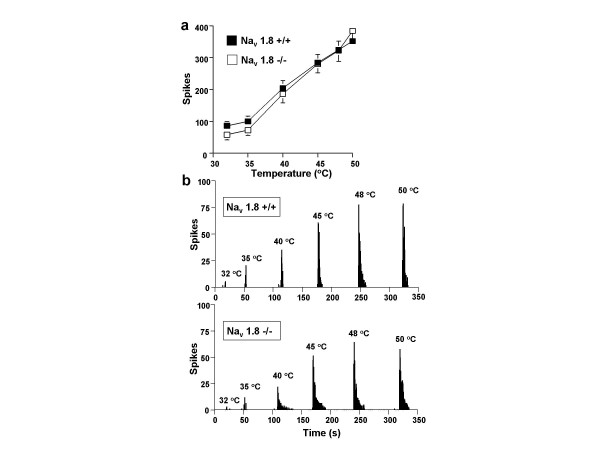
**Na_v _1.8-null mice show no alteration in thermally-evoked dorsal horn neuronal activity**. (a) Wide dynamic range neurones recorded from Na_v _1.8 -/- (n = 30) show similar activity to thermal stimuli compared to littermate control Na_v _1.8 +/+ (n = 45) mice. Data is expressed as means + or - sem. (b) Example traces of two single neurones, one recorded from a Na_v _+/+ mouse and one from a Na_v _-/- mouse. Thermal stimuli (water jet) were applied for 10 s onto the peripheral receptive field of the neurone located on the hindpaw. In both groups a graded response is displayed to increasing temperature and this is comparable in Na_v _1.8 -/- and Na_v _1.8 +/+ mice.

**Figure 3:**
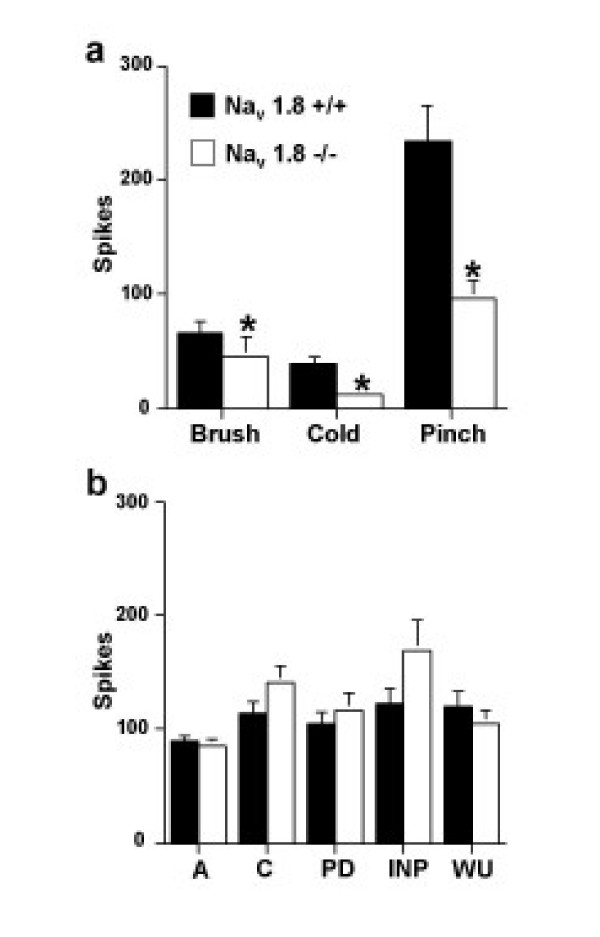
**Na_v _1.8-null mice show altered neuronal activity to in a stimulus-dependent manner**. (a) Wide dynamic range neurones recorded from Na_v _1.8 -/- (n = 30) show significantly reduced activity to brush, noxious cold 1°C and noxious pinch stimuli compared to littermate control Na_v _1.8 +/+ (n = 45) mice. Stimuli were applied to the peripheral receptive field of the neurone located on the hindpaw (b) Wide dynamic range neurones recorded from Na_v _1.8 -/- (n = 30) and Na_v _1.8 +/+ (n = 45) mice show no difference in responses evoked to transcutaneous electrical stimulation of the neuronal receptive field. Similar levels of neuronal activity evoked by A-fibres (A) and C-fibres (C), and related postdischarge (PD), input (INP) and wind-up (WU) measures were recorded (see methods for descriptions). Data is expressed as means + or - sem. *p < 0.05.

Neuronal responses evoked to natural stimuli were altered in mice lacking Na_v _1.8 compared to littermate controls. Nav 1.8 -null mice show marked deficits in mechanical coding compared to control (Figure 1) and this was found to be significant over both the non-noxious and noxious range (p < 0.05). Interestingly no such deficits were apparent for the neuronal coding of thermal stimuli to warm and noxious heat (Figure 2) recorded at the same time from the same neurones. Nav1.8-null mice also show a statistically significant reduction in their dorsal horn neuronal activity to brush, noxious cold and pinch stimuli (p < 0.05), which was more marked for the higher intensity pinch modality (Figure 3a).

In contrast, the evoked responses to transcutaneous electrical stimulation of the peripheral receptive field showed no difference between groups in either the threshold for activation of A-fibre (0.03 ± 0.004 and 0.06 ± 0.02 mA respectively) and C-fibre afferents (0.4 ± 0.08 and 0.37 ± 0.11 mA respectively), nor in their evoked neuronal responses, and related postdischarge, input and wind-up measurements (Figure 3b). Interestingly, the occurrence (20%; 6 neurones out of 30) and rate of ongoing spontaneous firing was lower in Na_v _1.8 -/- mice (2.9 ± 1.5 Hz) compared to littermate control (53%; 24 out of 45 neurones 4.11 ± 0.97 Hz), yet this was found to be significant only for the occurrence (p = 0.004, Fisher's Exact Test).

## Discussion

This study clearly shows that mice lacking the VGSC Na_v _1.8 have marked deficits in the evoked activity of spinal dorsal horn neurones in response to mechanical stimuli, whether it be brush, von Frey or pinch. Further, the more noxious the stimulus, the greater the deficit. Given the reported distribution of Na_v _1.8 channels predominantly in small diameter nociceptive fibres over non-noxious stimuli-sensing large diameter fibres, the comparative reductions fit well with the location of the channel on mainly noxious small-diameter afferents. No such reductions in neuronal activity were determined to thermal stimuli over the warm and noxious heat range. Thus deletion of Na_v _1.8 reveals a stimulus-defined role for this VGSC in the transmission of sensory stimuli from the periphery to the spinal cord via predominantly nociceptive primary afferents as its distribution dictates. It may be that Nav 1.8 VGSCs are localised to a population of mechanosensitive fibres, or possibly associated with another transducer responsible for mechanosensation.

The in vivo neuronal responses presented here are in line with behavioural studies where Na_v _1.8-null mice were shown to display deficits in responses to noxious mechanical pressure, yet not to thermal stimulation [4,8]. These studies did not however report an increase in withdrawal threshold to von Frey filaments. However this apparent discrepancy can be attributed to the different measures made in behavioural and electrophysiological assessments. Behavioural testing can only assess von Frey intensities up to the threshold for withdrawal, whereas, in anaesthetised animals here we have studied responses to both sub- and suprathreshold stimuli. Therefore we are able to demonstrate these marked reductions in the response to stronger mechanical stimulation at the level of the second order spinal cord neurones which behavioural testing is unable to reveal.

In response to electrical stimulation of the neuronal peripheral receptive field, Na_v _1.8 -/- and Na_v _1.8 +/+ mice exhibited similar levels of activity. Further the related input, post-discharge, and wind-up responses were also unaltered. Each of these electrical measures can be related to a specific part of the nociceptive pathway. The non-potentiated input response reflects the level of synaptic transmission between the central terminals of primary afferents and the neurones of the spinal cord dorsal horn. Although Na_v _1.8 is expressed mainly in small diameter primary afferents [2,3] and as Na_v _1.8 currents are localised to the peripheral nerve terminals [9], the fact that C-fibre evoked dorsal horn neuronal responses were unaltered in the knockout mice is not unexpected since electrical stimulation directly activates the primary afferent peripheral terminals independent of peripheral transduction mechanisms located at these sites. Furthermore, C-fibres also express a number of other VGSCs [10] which would also be activated by electrical stimulation, therefore potentially minimising the impact of Na_v _1.8 deletion. Postsynaptic, spinal NMDA receptor-mediated post-discharge and wind-up measurements were also not altered in Na_v _1.8-null mice. These measures are indicative of central sensitisation and neuronal hyperexcitability, and since Na_v _1.8 is not expressed within the spinal cord, its deletion should not affect these measures, as we have shown.

Given that VGSCs underlie action potential generation and thus determine neuronal excitability it could be argued that absence of Na_v _1.8 would inherently cause a global reduction in the activity of sensory neurones lacking native expression, and indeed the occurrence but not rate of ongoing spontaneous activity was lower in Na_v _1.8-null mice seen here and similarly reported in damaged sensory axons [11]. However, no difference was observed between mice expressing or lacking Na_v _1.8 in neuronal responses to warm and noxious heat and electrical stimulation therefore stimulus-evoked neuronal excitability is not globally affected by this genetic modification. This also emphasises the importance of peripheral terminals as sites responsible for the selective deficits in mechanosensitivity seen in Na_v _1.8-null mice.

A potential complication in interpretation of these results could be compensatory changes in other proteins, a common problem with all genetic deletion studies. It is reported that in the Na_v _1.8 knockout there is increased expression of TTX-sensitive currents, but not TTX-resistant, possibly due to an increase in Na_v _1.7 [2]. Importantly, in the Na_v _1.8 null mouse we observed a *deficit *in neuronal responses to peripheral mechanical stimulation. Any functional compensatory upregulation of Na_v _1.7, for example, impacting upon the phenotype, would increase neuronal activity, yet we still observed a clear selective reduction in this modality. Deletion of Na_v _1.8 must therefore underlie the reduction in mechanically evoked neuronal responses. The balance of effect of Na_v _1.8 deletion and compensatory increases may actually be masking less prominent roles for Na_v _1.8 in the transmission of thermal stimuli.

The neuronal profile generated by deletion of Na_v _1.8 does not resemble the neuronal phenotype of nociceptive -specific Na_v _1.7 knockout mice, which in contrast reveal no alteration in dorsal horn neuronal responses to the same mechanical and thermal stimuli as used here [12]. Behaviourally these mice are resistant in the Randall-Selitto mechanical pressure test, and also show striking deficits in the development of inflammatory pain. This strongly suggests that the results presented here are attributable to the VGSC deleted.

## Conclusion

We conclude that deletion of Nav 1.8 results in significantly reduced dorsal horn neuronal responses in a stimulus-dependent manner, such that mechanical, but not heat, stimuli are affected. This is not due to a general reduction in the excitability of primary afferent fibres, but more likely a result of disruption to the mechanosensing apparatus located on these peripheral terminals. Given the major clinical importance of mechanical pain and the lack of any clear present data on the nature of the transducer(s) further studies on the exact roles and the development of blockers of Nav 1.8 will be important.

## Methods

Anaesthesia was briefly induced with 3% halothane (66% N2O and 33% O2), following which the mice were injected with urethane (240 mg/kg). Animals were placed in a stereotaxic frame and a laminectomy was performed to expose the L3–L6 spinal segments. Extracellular recordings were made from single wide dynamic range neurones using parylene coated tungsten electrodes (A-M Systems, USA). Neurones, visualised on an oscilloscope, were isolated and discriminated on a spikes amplitude and waveform basis. All neurones had receptive fields over the hindpaw. Depth of the neurone was measured from the surface of the spinal cord. On isolation of a neurone any spontaneous activity quantified over a period of at least 100 s, in the absence of any evoked stimulus.

Electrical stimulation was given via two needles inserted into the receptive field and the evoked activity was characterised and quantified as follows. A train of 16 stimuli was given (2 ms pulse duration, 0.5 Hz at three-times the C-fibre threshold), and the evoked neuronal responses were superimposed and separated – based on fibre conduction velocity and the resulting latency – into total A-fibre-evoked (0 – 50 ms), and C-fibre-evoked (50 – 250 ms) action potentials. Neuronal responses occurring after the C-fibre latency band of the neurone (250 – 800 ms) were classed as postdischarge, a result of repeated stimulation leading to wind-up neuronal hyperexcitability. The 'input' (non-potentiated response), and the 'wind-up' (potentiated response, evident by increased neuronal excitability to repeated stimulation) were calculated. Input = (action potentials (50 – 800 ms) evoked by first pulse at 3 times C-fibre threshold) × total number of pulses (16). Wind-up = (total action potentials (90 – 800 ms) after 16 train stimulus at 3 time C-fibre threshold) – Input.

A wide range of natural stimuli, including brush, von Frey filaments (1, 2, 5, 9, 15, 30, 75 g), heat (32, 35, 40, 45, 48 and 50°C water jet), were applied to the receptive field for 10 seconds per stimulus and the evoked neuronal firing was quantified. Data was captured and analysed by a CED 1401 interface coupled to a Pentium computer with Spike 2 software (Cambridge Electronic Design; PSTH and rate functions). The receptive field area was mapped on standard diagrams of the projected area of the plantar surface of the paw, to a pinch stimulus. Diagrams were copied to plain copier paper (80 g/m^2^) and marked areas were cut out and weighed. Receptive field sizes were measured as the weight of the particular area and expressed as a percentage of the mean weight of 10 control diagrams of the whole paw (79.0 ± 0.5 mg).

Data are presented as mean ± S.E.M. A Mann Whitney was used for statistical analysis and the level of significance was taken to be *p < 0.05. Unless otherwise stated.

## Abbreviations

**VGSC(s) **– Voltage Gated Sodium Channel(s)

## Authors' contributions

EM carried out the in vivo electrophysiology and drafted the manuscript. JW conceived of the study and provided the transgenic mice. EA and AD participated in the design of the study. All authors read and approved the final manuscript.

## References

[B1] Catterall WA (2000). From ionic currents to molecular mechanisms: the structure and function of voltage-gated sodium channels. Neuron.

[B2] Akopian AN, Sivilotti L, Wood JN (1996). A tetrodotoxin-resistant voltage-gated sodium channel expressed by sensory neurons. Nature.

[B3] Djouhri L, Fang X, Okuse K, Wood JN, Berry CM, Lawson SN (2003). The TTX-resistant sodium channel Nav1.8 (SNS/PN3): expression and correlation with membrane properties in rat nociceptive primary afferent neurons. J Physiol.

[B4] Akopian AN, Souslova V, England S, Okuse K, Ogata N, Ure J, Smith A, Kerr BJ, McMahon SB, Boyce S, Hill R, Stanfa LC, Dickenson AH, Wood JN (1999). The tetrodotoxin-resistant sodium channel SNS has a specialized function in pain pathways. Nature Neuroscience.

[B5] Kerr BJ, Souslova V, McMahon SB, Wood JN (2001). A role for the TTX-resistant sodium channel Nav 1.8 in NGF-induced hyperalgesia, but not neuropathic pain. Neuroreport.

[B6] Crabbe JC, Wahlsten D, Dudek BC (1999). Genetics of mouse behavior: interactions with laboratory environment. Science.

[B7] van der Staay FJ, Steckler T (2001). Behavioural phenotyping of mouse mutants. Behav Brain Res.

[B8] Nassar MA, Levato A, Stirling LC, Wood JN (2005). Neuropathic pain develops normally in mice lacking both Nav1.7 and Nav1.8. Mol Pain.

[B9] Brock JA, McLachlan EM, Belmonte C (1998). Tetrodotoxin-resistant impulses in single nociceptor nerve terminals in guinea-pig cornea. J Physiol.

[B10] Wood JN, Boorman JP, Okuse K, Baker MD (2004). Voltage-gated sodium channels and pain pathways. J Neurobiol.

[B11] Roza C, Laird JM, Souslova V, Wood JN, Cervero F (2003). The tetrodotoxin-resistant Na+ channel Nav1.8 is essential for the expression of spontaneous activity in damaged sensory axons of mice. J Physiol.

[B12] Nassar MA, Stirling LC, Forlani G, Baker MD, Matthews EA, Dickenson AH, Wood JN (2004). Nociceptor-specific gene deletion reveals a major role for Nav1.7 (PN1) in acute and inflammatory pain. Proc Natl Acad Sci U S A.

